# RNA-Seq reveals miRNA role in thermogenic regulation in brown adipose tissues of goats

**DOI:** 10.1186/s12864-022-08401-2

**Published:** 2022-03-07

**Authors:** Xin Liu, Yuehua Zhu, Siyuan Zhan, Tao Zhong, Jiazhong Guo, Jiaxue Cao, Li Li, Hongping Zhang, Linjie Wang

**Affiliations:** Farm Animal Genetic Resources Exploration and Innovation Key Laboratory of Sichuan Province, College of Animal Science and Technology, Sichuan Agricultural University, Chengdu, 611130 Sichuan People’s Republic of China

**Keywords:** microRNA, Brown adipose tissue, Goat, miR-433, Thermogenesis

## Abstract

**Background:**

MicroRNAs (miRNAs) are a family of short non-coding RNA molecules and play important roles in various biological processes. However, knowledge of the expression profiles and function of miRNAs on the regulation of brown adipose tissue (BAT) thermogenesis remains largely unknown.

**Results:**

In this study, we found that brown adipose tissue (BAT) existed within the perirenal fat at 1 day after birth (D1) and transferred into white adipose tissue (WAT) at 30 days after birth (D30) by UCP1 protein expression and immunohistochemistry analysis. After that, we performed RNA sequencing on six libraries of goat BAT and WAT. A total of 238 known miRNAs and 1834 goat novel miRNAs were identified. Moreover, 395 differentially expressed miRNAs including 167 up-regulated and 228 down-regulated miRNAs were obtained in BAT. For the known BAT enriched miRNA, 30 miRNAs were enriched in goat BAT but not in mouse BAT. In addition, miR-433 was enriched in goat BAT but not in mouse BAT. Gain- and loss-of-function experiments reveal that miR-433 reduced the lipid accumulation of brown adipocytes and decreased the expression of BAT marker and mitochondrial related genes. However, miR-433 had no effect on lipid accumulation and thermogenesis in white adipocytes. In addition, miR-433 inhibited the expression of MAPK8 by targeting to the 3’UTR of MAPK8 gene. These data demonstrate that miR-433 acts as a negative regulator in controlling brown adipocytes differentiation and thermogenesis.

**Conclusion:**

The present study provides a detailed miRNAs expression landscape in BAT and WAT. Furthermore, we found that miR-433, which was highly expressed on BAT had a negative regulatory function on the thermogenesis and adipogenesis in goat brown adipocytes. This study provides evidence for understanding the role of miRNAs in regulating BAT thermogenesis and energy expenditure in goats.

**Supplementary Information:**

The online version contains supplementary material available at 10.1186/s12864-022-08401-2.

## Background

In mammals, adipose tissue is classified into three types: brown adipose tissue (BAT), white adipose tissue (WAT), and beige adipose tissues. Compared with WAT for energy storage, BAT has a function that non-shivering thermogenesis is implemented by consuming multilocular lipid droplets, which is important for surviving of neonatal mammals [1, 2]. In addition, beige adipose tissues can be recruited to undergo thermogenesis under specific conditions. For example, prolong treatment with β3 agonist CL316243 causes WAT browning by abundantly increase of *UCP1*, *COX4*, and *PRDM16* [3]. Physical intervention also induces WAT browning such as cold exposure and exercise. When beige adipose tissue is recruited, it uses abundantly of blood glucose and fatty acids in the circulatory system for thermogenesis, so it has therapeutic potential for obesity and type 2 diabetes [4–6].

There are two ways of thermogenesis, one is the classical sympathetic-dependent norepinephrine (NE)-cAMP pathway, which stimulates skeletal muscle contraction to produce shivering thermogenesis [7]; the other is UCP1-independented mechanism that relies on BAT for non-shivering thermogenesis [8]. In sheep, BAT is abundantly present in the perirenal fat depots after birth, and generates large amounts of heat rapidly, and the expression of the UCP1 also reaches its peak at birth [9]. However, BAT rapidly decreases and largely disappears at 1 month of age, UCP1 expression is also gradually declined [10]. For neonatal mammals, the thermogenic capacity of BAT is important for maintaining normal body temperature [11]. Previous studies of BAT mostly focused on humans and mice, and less on farm animals. In fact, BAT is also an important organ of thermogenesis in lambs. BAT generate heat to 10% of the total amount of heat per day and increases lambs’ body temperature by 1 °C above adult sheep [12]. In recent study, we found that BAT is abundant within the goat perirenal fat at birth [13]. Therefore, BAT plays a critical role in reducing mortality, and explore how BAT is regulated in the newborn lambs to adapt to the cold extrauterine environment is necessary.

miRNAs are a family of short non-coding RNA molecules [14, 15] as an important and multifunctional regulatory factor in BAT. miRNAs like miR-32 [16], miR-155 [17], and miR-327 [18], are confirmed that they are involved in brown adipogenesis. Profit from the development of RNA sequencing technologies, an increasing number of miRNAs regulating BAT development and function have been identified. Zhang et al. screened miRNAs differentially regulated in mouse BAT between newborn (postnatal day 1–2) and adult (8-week-old) by sequencing miRNAs [19]. Tao et al. found that several miRNAs are differentially expressed between BAT and inguinal WAT of mice under cold stimulation and room temperature [20]. Güller et al. derived miRNAs potentially regulating BAT by comparing differentially expressed miRNAs between BAT and WAT by RNA sequencing [21]. However, few studies have investigated the changes of miRNAs that regulate BAT development in goat. In this study, we screened out the differentially expressed miRNAs between WAT and BAT by RNA sequencing, leading to a better understanding of miRNAs with regulatory functions in goat BAT development. Furthermore, we found that miR-433, which was highly expressed on BAT, had a negative regulatory function on the thermogenesis and adipogenesis in goat brown adipocytes.

## Results

### Characteristics of goat perirenal fat at 1 day (D1) and 30 days (D30) after birth

**Fig. 1:**
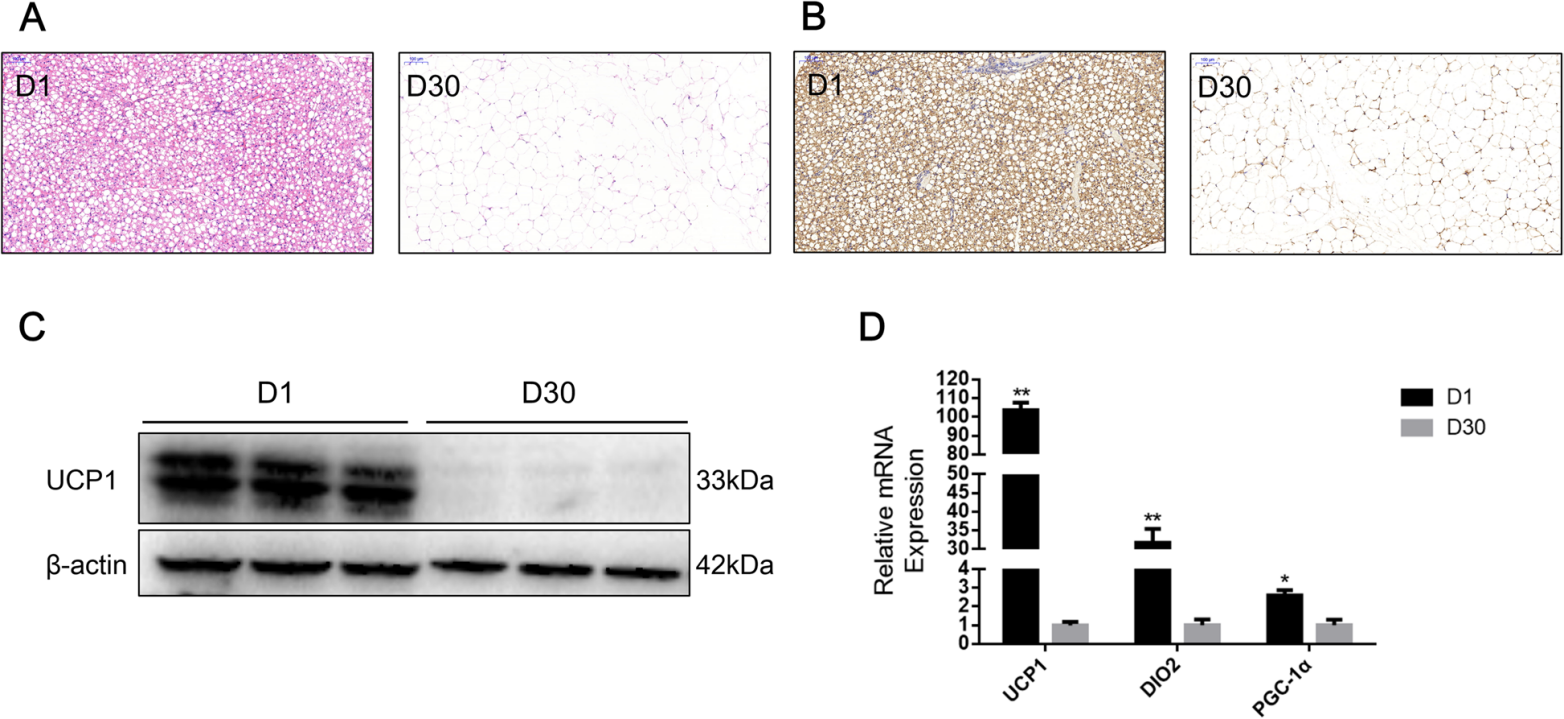
Characteristics of goat perirenal fat at D1 and D30. **A** Histological sections stained with hematoxylin; **B** UCP1 protein immunostaining from perirenal fat in D1 and D30; **C** Western blotting of UCP1 protein between D1 and D30 in perirenal fat; **D** Expression levels of UCP1, DIO2, and PGC1α were determined by qPCR. Error bars represent standard error of mean (SEM), *n* = 3, * *P* < 0.05, ** *P* < 0.01

To visualize and characterize the perirenal adipose tissue of 1 day after birth (D1) versus at 30 days after birth (D30), we observed the morphological differences by HE-stained sections. Compared to perirenal fat at D30, perirenal fat at D1 has smaller lipid droplets with a typical multilocular structure, but perirenal adipose tissue had larger lipid droplets at D30 (Fig. 1A). In addition, we observed an enrichment of UCP1 protein in perirenal fat at D1 by immunohistochemistry analysis (Fig. 1B). UCP1 protein was most abundant in perirenal adipose tissue at D1 and undetectable at D30 (Fig. 1C and Fig. S1). Subsequently, we performed an assay for marker genes of BAT. The mRNA levels of *UCP1*, *PGC1-α*, and *DIO2* were highly expressed at D1 (Fig. 1D). Those results indicated that BAT existed in newborn and dynamically transferred into WAT at D30.

### Small RNA sequencing and annotation in WAT and BAT

**Fig. 2:**
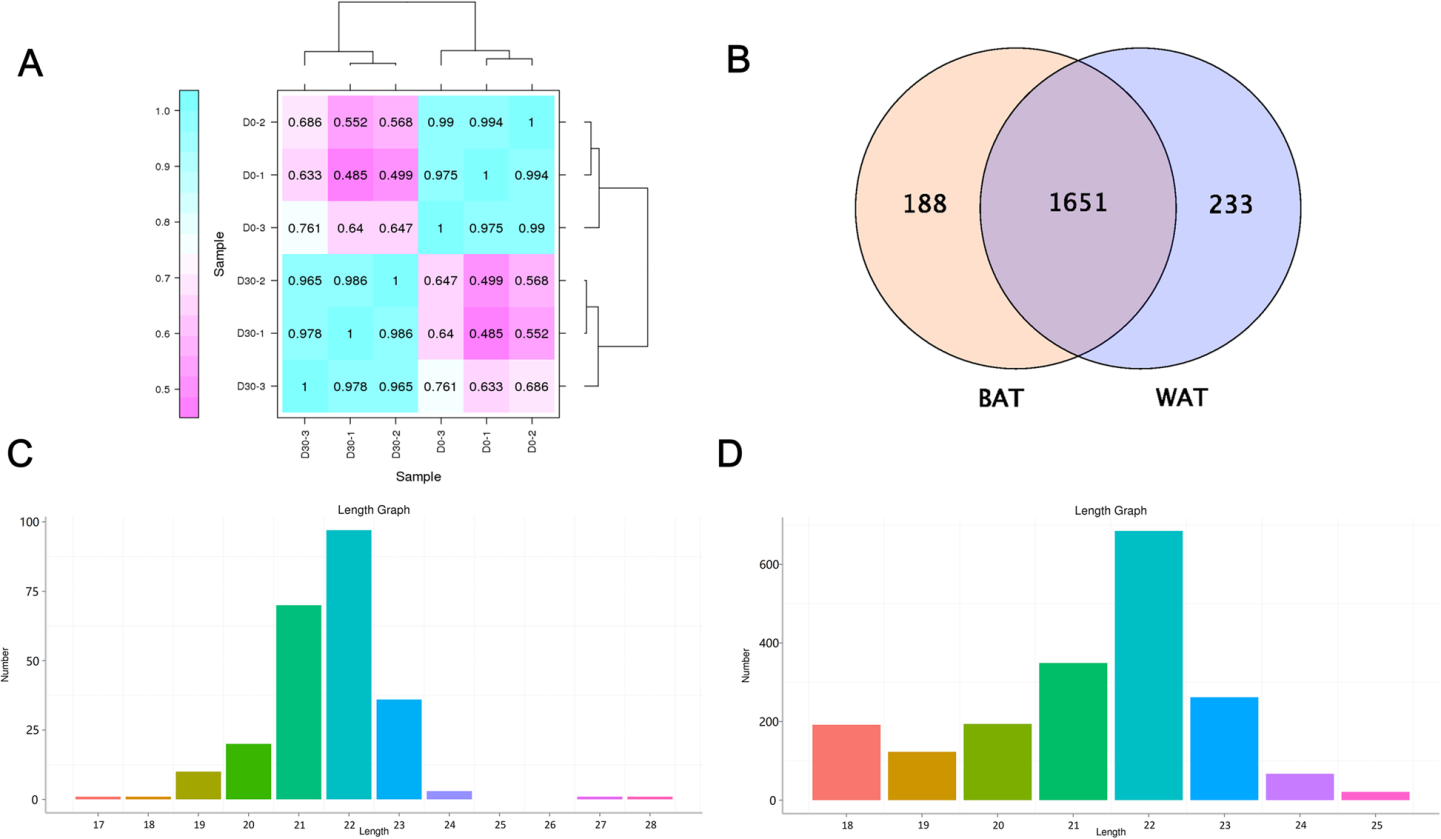
Small RNA sequencing and annotation in WAT and BAT. **A** Heat map of replicate samples of the same stage of goat perirenal fat. The color spectrum, ranging from white to blue, represents Pearson correlation coefficients; **B** Venn diagram showing miRNAs identified in BAT and WAT; **C** Length distribution of known miRNA; **D** Length distribution of novel miRNA

To identify key miRNAs that differentially regulate BAT and WAT, a total of 6 small RNA libraries were constructed from 6 individuals in two stages. Then, all the low-quality reads, containing poly-N reads, and sequences with less 18 nt or greater 30 nt were eliminated from corresponding raw reads. Then, a total of 65,731,042 clean reads were obtained and the average Q 30 score of the 6 libraries were over 98% (Table S1). The high inter sample correlation at different stages indicates excellent biological reproducibility of our samples from the same period (Fig. 2A). After filtering the rRNA, tRNA, and snoRNA, the remaining unannotated reads were used to identify miRNAs (Table S2). In addition, 61.60–67.79% of the unannotated reads per library were mapped to the goat reference genome (Table S3). We found a total of 2072 miRNAs containing 238 known miRNAs and 1834 novel miRNAs in the 6 libraries (Table S4). A total of 1651 miRNAs were found in both WAT and BAT. In addition, 188 miRNAs were specifically expressed in BAT and 233 miRNAs were specifically expressed in WAT (Fig. 2B). The length of miRNA was most in the range 21 nt to 23 nt, and the 22 nt was dominant (Fig. 2C, D), this is accord with typical miRNA length characteristics.

### Identification of differentially expressed miRNAs between WAT and BAT

**Fig. 3:**
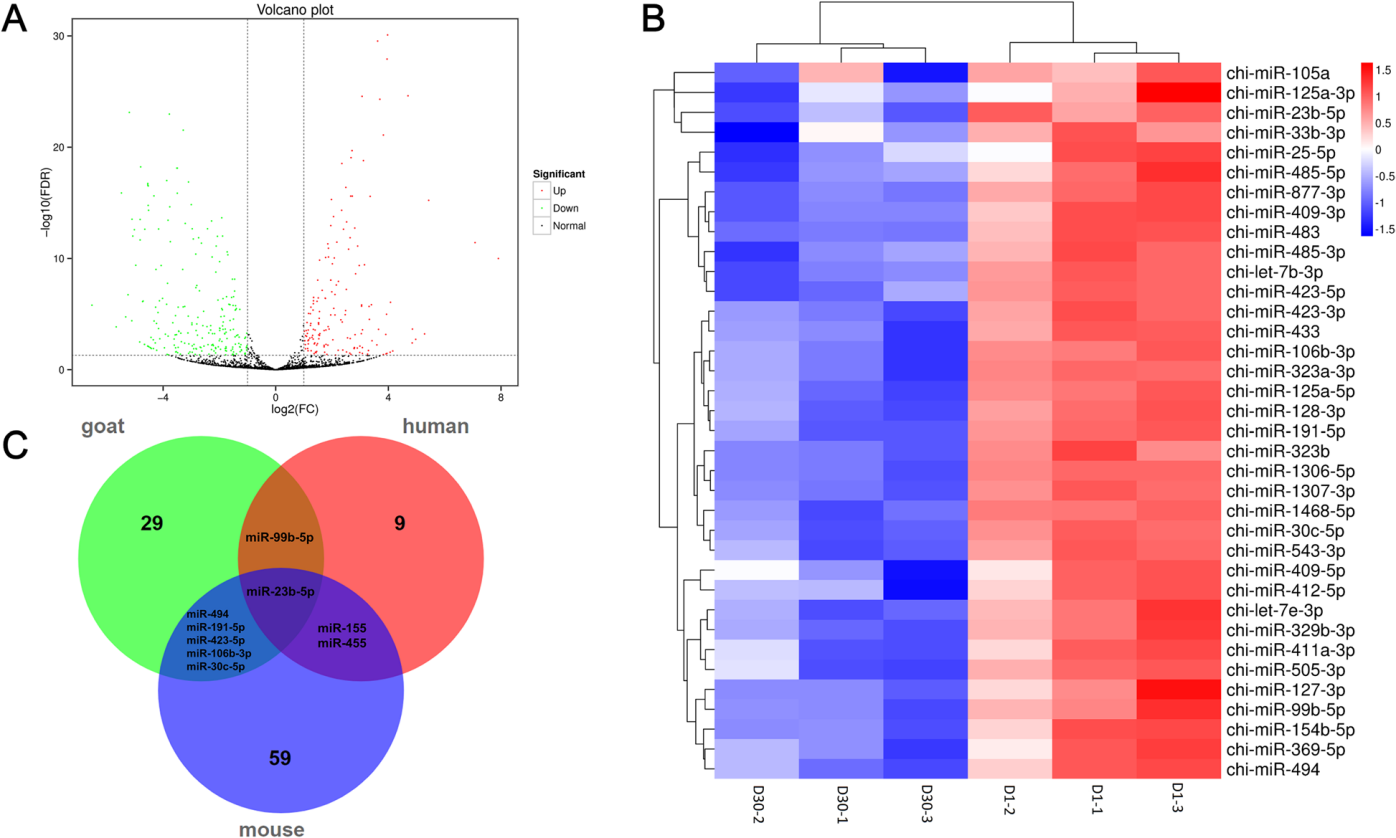
Identification of differentially expressed miRNAs between WAT and BAT. **A** Volcano plot showing differential miRNA expression profiles of BAT and WAT; **B** Hierarchical clustering Heatmap of samples with known BAT upregulated miRNAs; **C** Comparison of BAT enriched miRNAs in goat, human, and mouse BAT

To understand miRNA expression patterns in different adipose tissues, we performed differential expression analysis of miRNAs between BAT and WAT. The 395 differentially expressed miRNAs (DEmiRNAs) including 167 up-regulated and 228 down-regulated DEmiRNAs were obtained between BAT and WAT (Table S5) and shown in the volcanic plot (Fig. 3A). Cluster analysis indicated that BAT and WAT known DEmiRNAs were well clustered into two classes, indicate that the samples had good uniformity among the three replicates (Fig. 3B).

Then, a comparison was made among the known BAT-enriched miRNAs common to goat, human and mouse BAT. As shown in Fig. 3C, a total of 29, 59, and 9 miRNAs were enriched in goat, mouse, and human BAT, respectively. Of the 5 miRNAs were enriched in goat and mouse BAT but not human BAT, while 2 miRNAs were enriched in human and mouse BAT but not goat BAT. miR-99b-5p was found to be commonly enriched in goat and human BAT but not mouse. miR-23b-5p was identified as commonly enriched in all three species (Table S6). These results suggest that the BAT-enriched miRNAs in this study may have specific functions in regulating goat BAT thermogenesis.

### Validation of miRNAs by qPCR

**Fig. 4:**
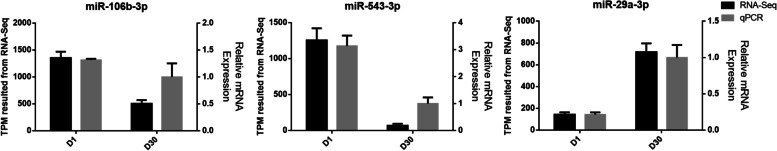
Validation of miRNAs by qPCR. The axis on the left represents transcripts Per Million (TPM) values of the miRNAs by RNA-seq, the axis on the right represents expression levels of miR-106b-3p, miR-543-3p, and miR-29a-3p by qPCR

To validate the accuracy of the sequencing data, three known miRNAs were selected to validate by qPCR analysis. It was shown that the expression levels of miR-106b-3p and miR-543-3p were significantly upregulated in perirenal fat at D1. In addition, miR-29a-3p was significantly upregulated in perirenal fat at D30 (Fig. 4). The qPCR expression trend of above miRNAs was almost consistent with the sequencing results, indicating that the sequencing results of the expression level of miRNAs were reliable.

### GO and KEGG pathway analysis for target genes of BAT enriched miRNAs

**Fig. 5:**
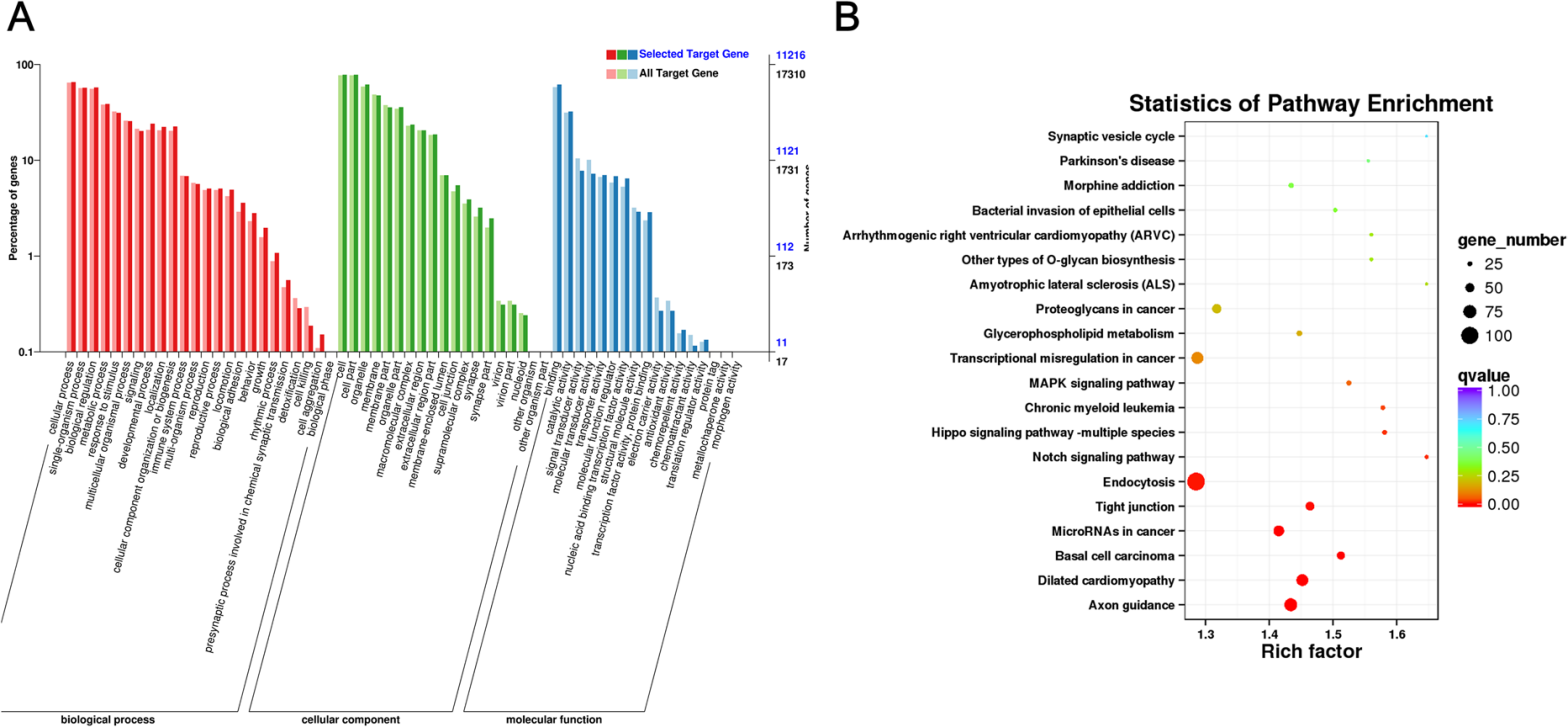
GO and KEGG pathway analysis for target genes of BAT enriched miRNAs. **A** Go analysis of BAT specific enriched miRNA target genes; **B** The top 20 enriched KEGG pathways for target genes of BAT upregulated miRNAs using the KEGG pathway database [22]

To understand regulatory functions of miRNA specifically expressed in BAT, we performed GO and KEGG pathway analysis for target genes of BAT enriched miRNAs (Table S7). These target genes were mainly enriched in functions cellular process, cell and binding functions (Fig. 5A). Next, we performed KEGG analysis (Fig. 5B), and the results showed that the target genes of these miRNAs were mainly involved in the Axon guidance, Endocytosis, Notch, Hippo, and MAPK signaling pathways (Table S8). These results suggest that BAT enriched miRNAs may play regulatory roles by targeting genes in these pathways.

### miR-433 prevents brown adipocyte differentiation and thermogenesis

**Fig. 6:**
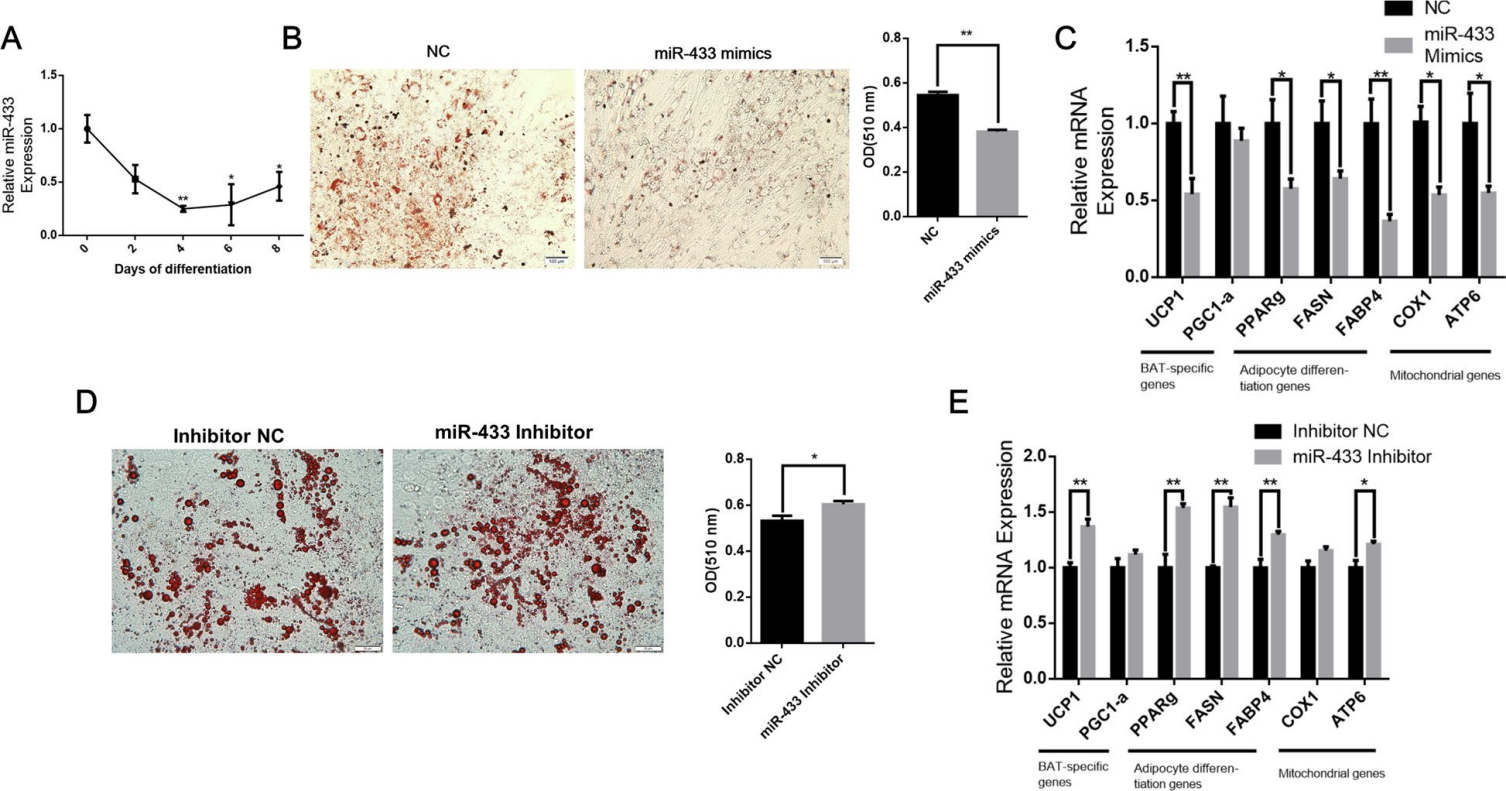
miR-433 prevents early differentiation of brown adipocytes. **A** Expression pattern of miR-433 during brown adipocytes differentiation; **B** Oil-Red-O staining of brown adipocytes transduced with mimics NC (left) or miR-433 mimics (right). The absorbance at 510 nm was detected; **C** qPCR analyses of gene expression in D4 of differentiated brown adipocytes transduced with mimics NC (black bars) or miR-433 mimics (gray bars); **D** Oil-Red-O staining of brown adipocytes transduced with inhibitor NC (left) or miR-433 inhibitor (right). The absorbance at 510 nm was detected; **E** qPCR analyses of gene expression in D4 of differentiated brown adipocytes transduced with inhibitor NC (black bars) or miR-433 inhibitor (gray bars). Error bars represent standard error of mean (SEM), *n* = 6, * *P* < 0.05, ** *P* < 0.01

Among BAT enriched miRNAs, miR-433 was one of the top 10 known miRNAs upregulated in BAT and little is known about the miR-433 function in BAT, we focused on miR-433 for further study. We found that the expression of miR-433 is highly expressed in proliferating brown preadipocytes and showed a downregulation trend with the differentiation of brown adipocytes (Fig. 6A). Then, miR-433 mimics were transfected into preadipocytes and overexpression of miR-433 reduced the lipid accumulation of brown adipocytes compared to NC mimics (*P* < 0.01) (Fig. 6B). In addition, miR-433 significantly decreased the expression of adipose differentiation marker genes such as *FASN* and *FABP4* (*P* < 0.05) (Fig. 6C). Furthermore, miR-433 significantly decreased *UCP1* expression, and mitochondrial related genes, including *COX1* and *ATP6* (*P* < 0.05) (Fig. 6C).

On the other hand, the silence of miR-433 increased the lipid accumulation of brown adipocytes (*P* < 0.05) (Fig. 6D) and increased the mRNA expression levels of several thermogenesis and adipogenesis markers including *UCP1*, *PPARg*, *FASN*, *FABP4*, and *ATP6* (*P* < 0.05) (Fig. 6E).

**Fig. 7:**
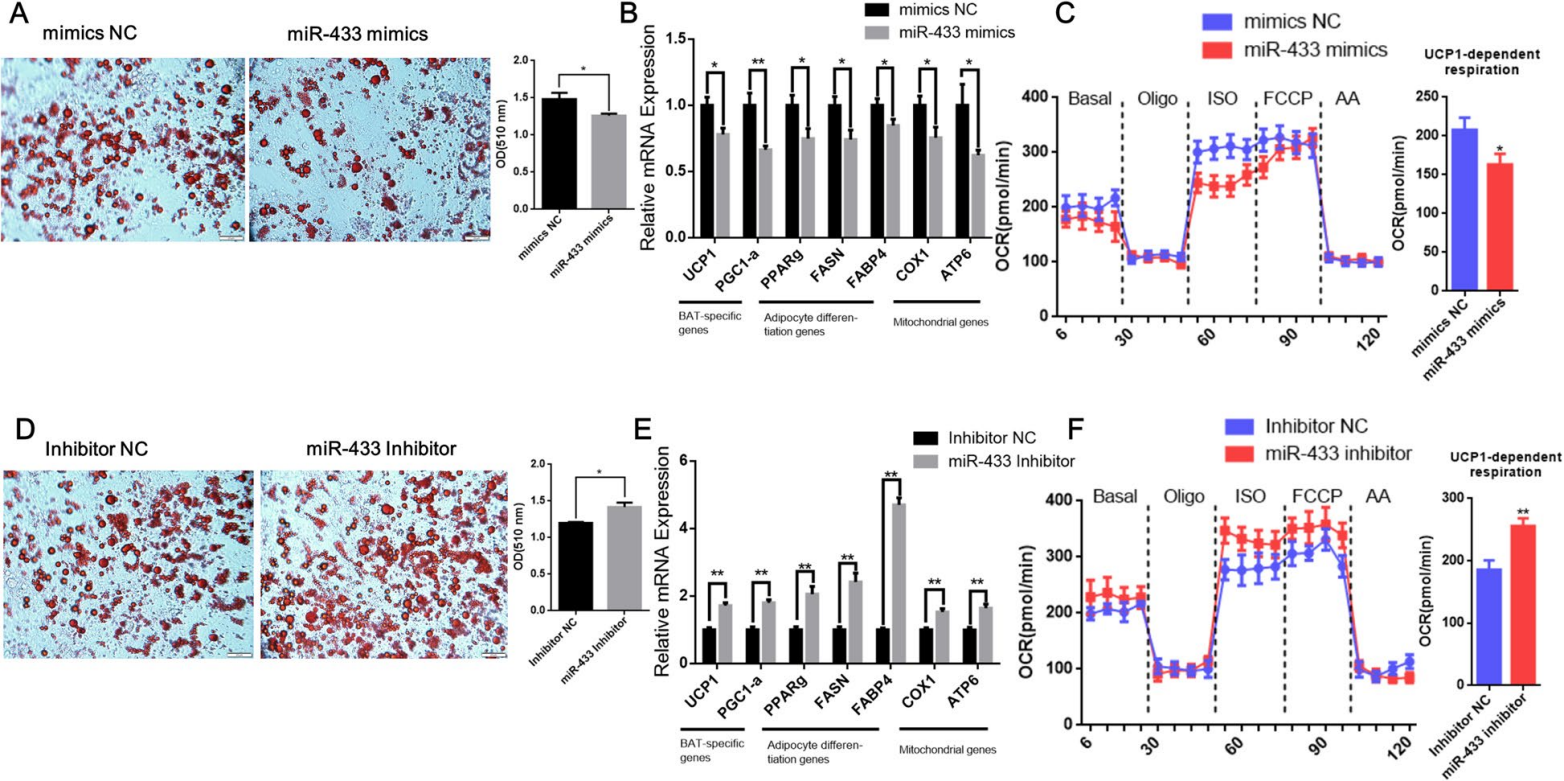
miR-433 prevents mature brown adipocyte differentiation and thermogenesis. **A** Oil-Red-O staining of brown adipocytes transduced with mimics NC (left) or miR-433 mimics (right) at D8 of differentiation. The absorbance at 510 nm was detected; **B** qPCR analyses of gene expression in mature brown adipocytes transduced with mimics NC (black bars) or miR-433 mimics (gray bars); **C** Oxygen consumption assay to mature brown adipocytes transduced with mimics NC or miR-433 mimics; ATP dependent respiration was inhibited by oligomycin, and UCP1 dependent respiration was activated by isoproterenol. UCP1-dependent oxygen consumption is measured by the highest recorded value after the injection of isoproterenol minus the lowest recorded rate after the injection of oligomycin. FCCP and antimycin A were used to determine the maximal and non-mitochondrial respiration, respectively. **D** Oil-Red-O staining of brown adipocytes transduced with inhibitor NC (left) or miR-433 inhibitor (right) at D8 of differentiation. The absorbance at 510 nm was detected; **E** qPCR analyses of gene expression in mature brown adipocytes transduced with inhibitor NC (black bars) or miR-433 inhibitor (gray bars); **F** Oxygen consumption assay to mature brown adipocytes transduced with inhibitor NC or miR-433 inhibitor. Error bars represent standard error of mean (SEM), *n* = 6, * *P* < 0.05, ** *P* < 0.01

To further verify whether miR-433 can regulate mature brown adipocytes, we transfected miR-433 mimics and harvest at D8 of differentiation. Our results showed that overexpression of miR-433 repressed oil red O staining (Fig. 7A) and decreased the mRNA levels of brown adipocyte and adipogenic factors, including *UCP1*, *PGC1-a*, *PPARg*, *FASN*, and *FABP4* (*P* < 0.05) in brown adipocytes (Fig. 7B). The expression levels of mitochondrial related genes, including *COX1* and *ATP6*, were also downregulated (*P* < 0.05) (Fig. 7B). We next performed oxygen consumption assay to assess whether miR-433 affects thermogenesis. As expected, UCP1 dependent oxygen consumption rate was markedly decreased in brown adipocytes with transfecting miR-433 mimics after the addition of isoproterenol (Fig. 7C). Subsequently, we tested whether silencing of miR-433 alters brown adipocytes differentiation. Knockdown of miR-433 with inhibitor increased brown adipocyte differentiation, as evidenced by the enhanced oil red O staining (Fig. 7D) and the upregulated mRNA levels of *UCP1*, *PGC1-a*, *PPARg*, *FASN*, *FABP4*, *COX1* and *ATP6* (*P* < 0.01) in brown adipocytes (Fig. 7E). UCP1 dependent oxygen consumption of miR-433-inhibitor-treated brown adipocytes were also significantly higher than NC-inhibitor-treated brown adipocytes after the addition of isoproterenol (Fig. 7F). Thus, our results illustrate that miR-433 prevents goat brown adipocyte differentiation and thermogenesis.

### miR-433 had no effect on differentiation and thermogenesis in white adipocytes

**Fig. 8:**
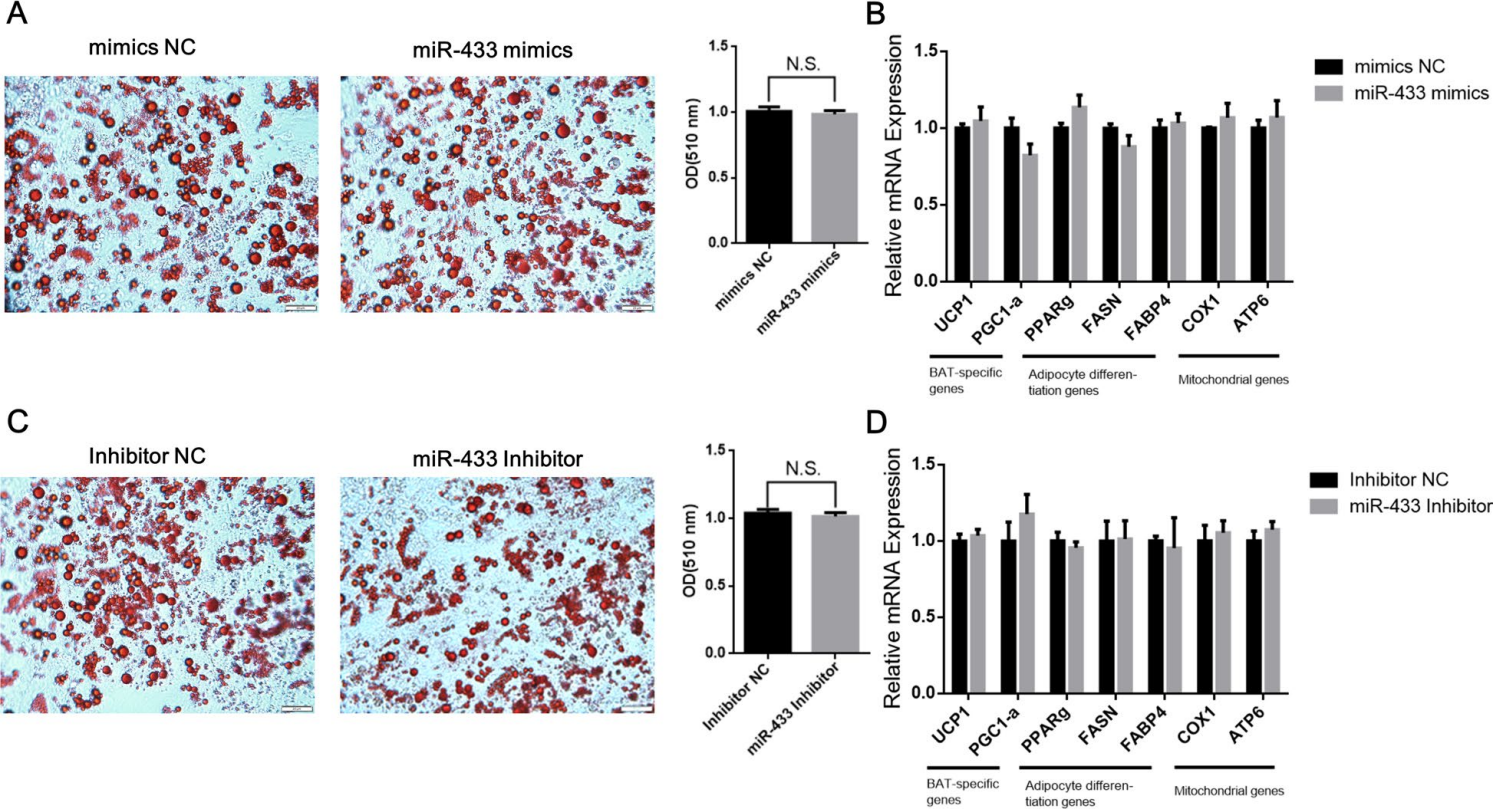
miR-433 had no effect on differentiation and thermogenesis in white adipocytes. **A** Oil-Red-O staining of white adipocytes transduced with mimics NC (left) or miR-433 mimics (right) at D8 of differentiation. The absorbance at 510 nm was detected; **B** qPCR analyses of gene expression in mature white adipocytes transduced with mimics NC (black bars) or miR-433 mimics (gray bars); **C** Oil-Red-O staining of white adipocytes transduced with inhibitor NC (left) or miR-433 inhibitor (right). The absorbance at 510 nm was detected; **D** qPCR analyses of gene expression in mature white adipocytes transduced with inhibitor NC (black bars) or miR-433 inhibitor (gray bars). Error bars represent standard error of mean (SEM), *n* = 6, * *P* < 0.05, ** *P* < 0.01

To investigate the role of miR-433 in the differentiation of white adipocytes, we transfected miR-433 mimics into white adipocytes. However, miR-433 mimics did not result in significant changes to lipid deposition (Fig. 8A). Adipogenic markers such as *PPARg*, *FASN*, and *FABP4* also did not show any significant changes (Fig. 8B). Furthermore, qPCR analysis of marker genes of brown adipocytes and mitochondria such as *UCP1*, *PGC1-a*, *ATP6*, and *COX1* showed that expression of these genes was not significantly changed (Fig. 8B). Next, we used miR-433 inhibitor to transfection. Silencing of miR-433 did not promoted the differentiation of white adipocytes (Fig. 8C). There is no change on the expression levels of brown adipocytes and mitochondria maker genes (Fig. 8D). These results suggested that miR-433 had no effect on white adipocyte differentiation and thermogenesis.

### *MAPK8* is a target gene of miR-433

**Fig. 9:**
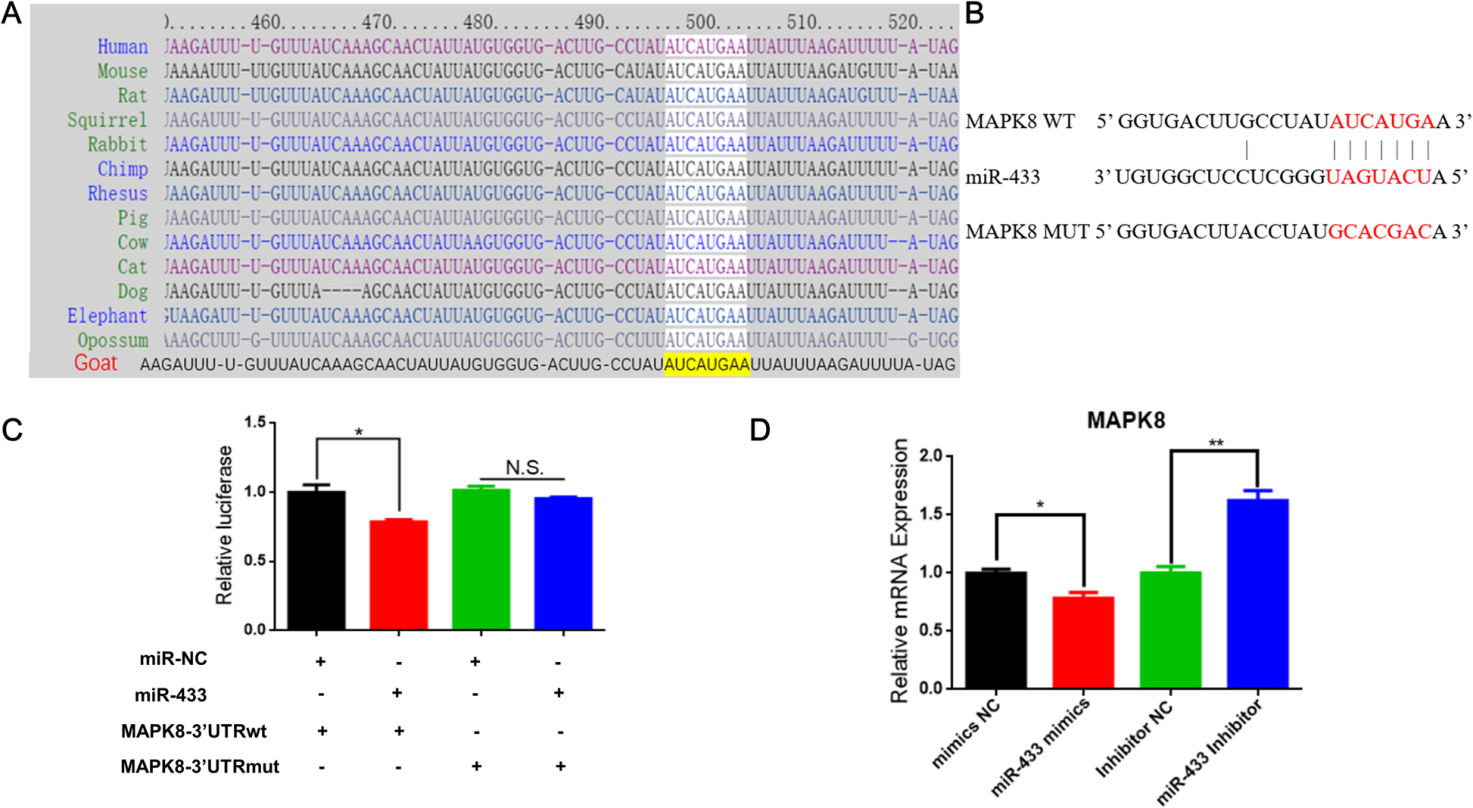
*MAPK8* is a target gene of miR-433. **A** The complementary sequence in the *MAPK* 3’UTR and the seed region of miR-433 are conserved among mammalian species; **B** Alignment details of miR-433 to *MAPK8* 3′ UTR binding site and seed region were mutated to generate *MAPK8* 3′ UTR Mut constructs. Red marks are seed region; **C** Luciferase reporter assays of Luc-*MAPK8*-WT and Luc-*MAPK8*-Mut in HEK293T cells show that miR-433 targets *MAPK8*; **D** Relative expression of *MAPK8* mRNA was detected using qPCR. Data are plotted as gene expression of miR-433 mimics group relative to mimics NC, and miR-433 inhibitors group relative to inhibitors NC group. Error bars represent standard error of mean (SEM), *n* = 6, * *P* < 0.05, ** *P* < 0.01

In the predicted targets with conserved sites, *MAPK8* was the 3th top hit using in silico prediction software targetscan. The program identified a highly probable miR-433 binding site within the 3′ UTR of *MAPK8* gene, which was also highly conserved in mammals from mice to goats and even human (Fig. 9A). To test whether *MAPK8* is a direct target of miR-433, we constructed the wildtype 3’UTR and mutation of *MAPK8* into psiCHECK2 vector (Fig. 9B). Transfection of miR-433 mimics led to a decrease in the activity of a luciferase reporter gene linked to the 3’UTR of goat *MAPK8* gene (Fig. 9C). Conversely, co-transfection with the *MAPK8* 3’UTR mutation did not change luciferase activity compared to the wildtype (Fig. 9C). The expression of *MAPK8* gene treated by miR-433 mimics or inhibitor was detected to be corresponded up or down regulated (Fig. 9D). The results indicate that miR-433 inhibited the expression of MAPK8 by targeting to the 3’UTR of MAPK8 gene.

## Discussion

Previous study in sheep have demonstrated that BAT is activated at birth, and then the highly expressed UCP1 rapidly stimulates non-shivering thermogenesis to maintain body temperature in cold environments [8]. After birth, the rectal temperature of the lamb is higher than that of the ewe, but it decreased rapidly to 32 °C to 35 °C [23]. The survival rate of lambs is compromised by heat loss even when the ambient temperature is mildly suitable [24]. To regulate body temperature normally, lambs may need a 15-fold increase in heat production to compensate for the heat lost to the environment [25]. Estimates of respiratory quotient suggest that the source of heat in newborn lambs after birth is primarily adipose tissue [26], whereas BAT is the major source of non-shivering thermogenesis in newborn lambs [27]. Previous studies have revealed that several miRNAs have a strong regulatory role for BAT thermogenesis in rodents [27] but have rarely been studied in large animals. In this study, we systematically identified differentially expressed miRNAs between goat BAT and WAT, and revealed several miRNAs that were associated with BAT development.

Previous studies have been explored the mechanism of BAT regulation by a variety of miRNAs using mice as a model. In this study, 167 BAT enriched miRNAs were obtained in goat BAT. Of the 6 miRNAs were found to be commonly enriched in goat and mouse BAT. Moreover, these BAT enriched miRNAs have already been reported to play important roles in BAT thermogenesis. For example, the miR-30c was confirmed to positively regulate BAT thermogenesis and differentiation by targeting the receptor-interacting protein 140 (RIP140) [28], whereas miR-191-5p, miR-494, and miR-106b-3p negatively regulated BAT development. Among them, miR-191-5p inhibition was shown to promoted the browning of WAT by targeting *PRDM16* [29]. miR-494 regulates mitochondrial biogenesis and thermogenesis by targeting *PGC-1α* [30]. And miR-106b is a negative regulator of BAT development and miR-106b knockdown induces the expression of BAT marker genes and promoted the lipid accumulation in brown adipocytes [31].

However, many miRNAs enriched in goat BAT were not enriched in mouse BAT. Interestingly, by KEGG pathway analysis, the target genes of goat BAT enriched miRNAs are associated with regulatory signaling pathways including the Notch, Hippo, and MAPK signaling pathways. The role of MAPK as a classical signaling pathway regulating BAT development and thermogenesis is well established [32]. In this study, we verified that *MAPK8* was a target gene of miR-433. MAPK8 is also known as JNK1. It was previously shown that JNK1 inhibition prevents cold-induced white adipose browning by inhibiting the JNK pathway, which impairs 3 T3-L1 cell differentiation, and decreases the expression levels of thermogenic genes [33]. MAPK kinase 6 (MKK6) is the upstream p38 activator and MKK6 deletion in mouse increases thermogenic capacity and browning of WAT [34]. In addition, miR-32 activates the expression of downstream thermogenic genes by inhibiting *TOB1* to drive the p38/MAPK pathway and then activate inguinal WAT browning by promoting the secretion of FGF21 [16]. Moreover, for the Notch signaling pathway, its downstream TLE3 could inhibit BAT formation by antagonizing PRDM16 binding to PPARγ in a competitive manner [35, 36]. In our results, TLE3 was targeted by several BAT enriched miRNAs including chi-miR-1307-3p, chi-miR-326-3p, chi-miR-423-3p, chi-miR-874-3p, and chi-miR-877-3p. The targeted genes of BAT enriched miRNAs were also involved in Hippo signaling pathway, including the targeted genes STK3 and STK4 of novel miR-320, novel miR-971, and novel miR-1073. Serine/threonine-protein kinase 3 (STK3) and STK4 are the two members in the Hippo signaling pathway. Genetic inactivation of STK3 and STK4 increases mitochondrial content and promotes mitophagy and energy expenditure in brown and beige adipose tissues [37]. These results indicate that these miRNAs may be involved in BAT thermogenesis through regulating their target genes.

Studies of single-nucleus RNA-sequencing have shown that BAT comprises adipocytes and preadipocytes, as well as endothelial cells, T cells, macrophages, fibroblasts, B cells, skeletal muscle cells, dendritic cells [38]. These other cell types may affect the results of miRNA expression files. We recognize that our research has limitation. Although we identified some BAT enriched miRNAs in goats, the expression file and roles of miRNAs underlying the cells subpopulation difference in BAT still need to be investigated in the future study.

In this study, miR-433 was enriched in goat BAT but not mouse BAT, while there is no report on the miR-433 function in BAT. Previous studies revealed that miR-433 plays a regulatory role in tissue fibrosis, it promotes renal fibrosis by targeting *AZIN1* to activate the TGF-β/SMAD3 signaling pathway [39]. Similarly, miR-433 is also regulated by the TGF-β/JNK1-AZIN1 axis to promote cardiac fibrosis [40]. In addition, miR-433 inhibits osteoblast differentiation by directly targeting Runx2 gene [41]. As a tumor suppressor, miR-433 strongly inhibits MHCC97H cell migration by targeting *CREB1* [42]. In this study, we revealed that miR-433 reduced the lipid accumulation of brown adipocytes and increased BAT marker and mitochondrial related genes. To our knowledge, miR-433 is first found to be a suppressor of brown adipocyte adipogenesis and thermogenesis.

## Conclusions

In summary, we compared differentially expressed miRNAs in BAT and WAT by RNA sequencing. For the BAT enriched miRNA, 30 miRNAs were enriched in goat BAT but not in mouse BAT. Among goat BAT enriched miRNAs, we validated that miR-433 negatively affects brown adipocytes differentiation and thermogenesis. This study will provide a reference and basis for future studies on BAT thermogenesis in goats. The role of miRNAs in regulating thermogenesis and energy expenditure in goats, however, requires further investigation.

## Methods

### Animal and sampling

All animals were raised at the breeding center of Sichuan Agricultural University, Ya’an, China (~ 1000 m altitude, 103.00°E, 29.98°N), annual temperature is 14 °C in average whereas humidity is 52%. For this study, the female Chuanzhong Black goats (10 ewes) were artificially inseminated with semen from one Ram. After that, we selected six female goat kids from three pairs of twins. Then, they were sampled in two periods (1 day and 30 days after birth, D1 and D30) respectively. Goat kids had water ad libitum and were fed breast milk with hay supplementation twice daily. All goats were fasted overnight before intramuscular injection with su mian xin (Shengda, Changchun, China) at a dose of 0.1 mL body weight. Under full anesthesia, all goats were sacrificed by arterial bleeding. Perirenal adipose tissue was collected and chilled with liquid nitrogen immediately, and stored at − 80 °C.

### Histology analysis

Perirenal adipose tissues were fixed with 4% paraformaldehyde and embedded in paraffin. Sections were stained with hematoxylin (HE). Adipose tissues morphology was photographed by Olympus BX-50F light microscope (Olympus Optical, Tokyo, Japan).

### Immunohistochemistry

Optimal cutting temperature (OCT) compound (Elkhart, IN, USA) was used for embedding adipose tissues, which snap frozen to − 80 °C. Then, it was cut into 5 μm thick sections with a cryostat (Leica, Bensheim, Germany), after which they were incubated with rabbit anti-UCP1 (absin, Shanghai, China) at a dilution of 1:500 overnight at 4 °C. The horseradish orseradish peroxidase-conjugated goat anti-rabbit IgG (Abclonal, Wuhan, China) was at a dilution of 1:2000 and incubated at 37 °C for 1 h. Streptavidin biotin complex (SABC) methods were employed to visualize. The Olympus BX-50F light microscope (Olympus Optical, Tokyo, Japan) was used to photograph the sections.

### Total RNA isolation

Total RNA was purified from perirenal fat with RNAiso Plus reagent (Takara, Tokyo, Japan). RNA integrity was assessed by the Agilent Bioanalyzer 2100 system (Agilent Technologies, CA, USA). The concentration of total RNA was assessed using a Nano Drop 2000 (Thermo, MA, USA).

### Small RNA library construction and sequencing

A total amount of 2.5 μg RNA was used for the RNA sample preparations. NEBNext® Ultra™ small RNA Library Prep Kit for Illumina® (NEB, MA, USA) was used to construct the sequencing library. Firstly, the 5′ and 3′ sRNA adaptor were ligated by T4 RNA ligase. Then the Ligation Reaction Buffer (2X) and Ligase Enzyme Mix were added to ligate the 5′ and 3′ sRNA adaptor. The mixed system was incubated in a thermal circulator at 25 °C for 1 h. Secondly, the first cDNA chain was synthesized by reverse transcription, and then PCR amplification and size selection were performed. PAGE gel was used for screening purpose of electrophoretic fragments, and the fragments were rubber cutting recycling as a small RNA library. Finally, all the libraries were sequenced on an Illumina NovaSeq 6000 platform.

### Classification and annotation of small RNAs

By Bowtie software [43], the clean reads were aligned with Silva [44], GtRNAdb [45], Rfam [46] and Repbase database [39] to filter ribosomal RNA (rRNA), transfer RNA (tRNA), small nuclear RNA (snRNA), small nucleolar RNA (snoRNA), small cytoplasmic RNA (scRNA) and repeats. Then unannotated reads were obtained and were mapped to the goat reference genome ARS1 to identify miRNAs. Finally, the remaining reads were used to detect known miRNA and novel miRNA predicted by comparing with known miRNAs from miRBase (v21) and miRDeep 2 (v2.0.1.2). Based on pre-miRNA sequence to identify whether miRNA is novel miRNA and Randfold software (v2.0) was used for novel miRNA secondary structure prediction.

### Differential expression analysis of miRNAs

The expression of miRNAs was normalized by TPM (transcripts per million) [40], and DESeq R package (1.8.3) was used to identify the differential expression miRNAs (DE miRNAs). To determine the significant DE miRNAs, two criteria were followed: (1) changes of expression level were more than or equal 2-fold (|log2(FC)| ≥ 1), FC (fold change); (2) adjusted *P*-value (FDR, false discovery rate) ≤ 0.05. DE miRNAs were clustered and visualized expression patterns by STEM software (v 1.1).

### Target gene annotation of miRNA

We employed miRanda [41] and targetscan (http://www.targetscan.org/) for target gene prediction of miRNAs. Annotation information of target genes was obtained by aligning predicted target gene sequences to NR (NCBI non-redundant protein sequences) [42], Swiss-Prot [47], GO [48], COG [49], KEG G[22], KOG [50], Pfam (Protein family) databases [51], eggNOG [52] using BLAST software.

### Brown preadipocytes cell isolation, culture and differentiation

Brown preadipocytes were isolated from perirenal adipose tissue of Chuanzhong black goats in D1. The tissue was washed repeatedly with PBS, and visible blood vessels and other tissues were removed. Tissues were digested with 2 mg/mL collagenase I (Sigma, MA, USA) for 25 min at 37 °C. After centrifugation at 1000 rpm/min, the supernatant was discarded and DMEM (10% fetal bovine serum, 2% penicillin/streptomycinat) was added, after which the cells were resuspended by pipetting, plated into culture flasks and placed in incubator (5% CO_2_, 37 °C). Until 100% confluence, to induce differentiation, DMEM was supplemented containing 5μg/mL of insulin (Solarbio, Beijing, China), 1 μM dexamethasone (Sigma, MA, USA), 1 μM 3-Isobutyl-1-methylxanthine (Sigma, MA, USA), 0.5 mM IBMX (Sigma, MA, USA), 1 μM Rosiglitazone (Sigma, MA, USA) and 1 nM triiodothyronine (Selleck, TX, USA). Two days later, the differentiation medium was changed to maintenance medium (DMEM supplemented with 10% FBS, 5 μg/ml of insulin and 1 nM triiodothyronine).

### White preadipocytes cell isolation, culture and differentiation

Primary white preadipocytes were isolated from perirenal adipose tissues of Chuanzhong black goats in D30 by 2 mg/mL collagenase I (Sigma, MA, USA) digestion for 70 min at 37 °C. Until 100% confluence, to induce differentiation, DMEM was supplemented containing 5 μg/mL of insulin (Solarbio, Beijing, China), 1 μM dexamethasone (Sigma, MA, USA), 1 μM 3-Isobutyl-1-methylxanthine (Sigma, MA, USA), 0.5 mM IBMX (Sigma, MA, USA), 125 μM Indomethacin (Selleck, TX, USA) and 1 nM triiodothyronine (Selleck, TX, USA). Two days later, the differentiation medium was changed to maintenance medium (DMEM supplemented with 10% FBS, 5 μg/ml of insulin and 1 nM triiodothyronine).

### Cell transfection and oil red O staining

miR-433 mimics, negative control mimics, miR-433 inhibitor, and negative control inhibitor (Sangon, Shanghai, China) were transfected by Lipofectamine 3000 (Invitrogen, CA, USA). After 4 days, lipid droplets were stained using Oil Red O solution (Sigma, MA, USA). After staining, 1 mL isopropanol was used to dissolve the oil red O dye, and the absorbance at 510 nm was detected.

### Quantitative real-time PCR

For miRNA qPCR, reverse transcription of miRNA was performed by mir-XTM miRNA First-Strand Synthesis Kit (Takara, Tokyo, Japan). Briefly, 1 μg of total RNA was incubated in a thermal cycler for 1 h at 37 °C, then terminating at 85 °C for 5 min to inactivate the enzymes. The qPCR was carried out with mixture of 0.8 μL of cDNA, 0.4 μL primers, 5 μL of Chamq Universal SYBR qPCR Master (Vazyme, Nanjing, China), and 3.8 μL ddH_2_O. And the reference gene *U6* was used to normalize the expression levels by the 2^-ΔΔCt^ method. For qPCR of target genes (Table S9), the cDNA of mRNA was obtained by Primer ScriptTM RT reagent Kit (Takara, Tokyo, Japan). The reference gene *TBP* [53] was used to correct gene expression levels. All data are expressed as the mean ± SEM. The one-way ANOVA was used in SPSS 19.0 (IBM, NY, USA), and Duncan’s new multiple range tests were used to analyze statistical significance.

### Western blotting

The total protein of cells was extracted according to the instructions of Bestbio Total Protein Extraction Kit (Bestbio, Shanghai, China). Proteins were transferred to PVDF membranes by Trans-Blot TurboTM (Bio-Rad, CA, USA). 5% fat free milk was used for blocking and the blocking conditions were 37 °C for 2 h. Antibodies were diluted 1:500 for rabbit anti-UCP1 (absin, Shanghai, China), 1:1000 for rabbit anti-β-actin (Abclonal, Wuhan, China). The PVDF membranes was incubated with primary antibody at 4 °C overnight, and the secondary antibody (HPR-labeled goat anti-rabbit IgG, 1:1000 dilution, Beyotime, Shanghai, China) was incubated at 37 °C for 1.5 h. Finally, an ECL detection system (Beyotime, Shanghai, China) was used to detect immunoreactive proteins.

### Oxygen consumption assays

Cells were seeded into 96 well plates and until the D4 of differentiation for transfection experiments. Four days after transfection, oxygen consumption rate was measured using the Seahorse XF96 Analyser (Agilent Technologies, CA, USA) following the protocols [54]. Baseline measurements of OCR were performed for 24 min before the addition of oligomycin. Oligomycin (5 μM) inhibits the activity of the ATP synthase, and injections of isoproterenol (0.5 μM) allowed measurement of UCP1-dependent uncoupled respiration. UCP1-dependent oxygen consumption is measured by the highest recorded value after the injection of isoproterenol minus the lowest recorded rate after the injection of Oligomycin. FCCP (1 μM) and antimycin A (5 μM) were used to determine the maximal and non-mitochondrial respiration, respectively.

### Luciferase reporter assays

We constructed the 3’UTR and 3’UTR mutation of *MAPK8* into psiCHECK2 vector. Pending that HEK293T cells reached 80% confluence, the vector and miR-433 were co transfected with Lipofectamine 3000 (Invitrogen, CA, USA). Cells were harvested two days after transfection. Luciferase activity was detected by using TransDetect® Double-Luciferase Reporter Assay Kit (Transgen, Beijing, China).

## Supplementary Information

**Additional file 1: Fig. S1.** Whole membrane images for Fig. 1C.

**Additional file 2: Table S1.** Sequencing data statistics.

**Additional file 3: Table S2.** Statistical table of sRNA classification annotation.

**Additional file 4: Table S3.** Comparison information of reference genome.

**Additional file 5: Table S4**. Information of all identified miRNAs.

**Additional file 6: Table S5.** Information of DEmiRNAs.

**Additional file 7: Table S6.** Comparison of BAT enriched miRNAs in mouse, human, and goat.

**Additional file 8: Table S7.** Prediction results of miRNA target genes.

**Additional file 9: Table S8.** Information of the enriched KEGG terms for BAT enriched miRNAs target genes.

**Additional file 10: Table S9.** Sequences of primer used for RT-qPCR.

## Data Availability

All data generated in this study are included in the main article and its supplementary files. All the raw sequencing data have been deposited in the NCBI Sequence Read Archive (SRA) database (Accession no. PRJNA750335).

## Abbreviations

miRNA: MicroRNA
BAT: Brown adipose tissue
WAT: White adipose tissue
D1: 1-day
D30: 30-days
MAPK: Mitogen-activated protein kinase
DEmiRNAs: Differentially expressed micro RNAs
TPM: Transcripts Per Million
FDR: False discovery rate
GO: Gene Ontology
KEGG: Kyoto Encyclopedia of Genes and Genomes
pre-miRNA: Precursor miRNA
rRNA: Ribosomal RNA
snoRNA: Small nucleolar RNA
snRNA: Small nuclear RNA
tRNA: Transfer RNA
scRNA: Small cytoplasmic RNA
