# Supplementary figures and images for: RNA-Seq reveals miRNA role in thermogenic regulation in brown adipose tissues of goats
Source: BMC Genomics. 2022 Mar 7;23:186. doi: 10.1186/s12864-022-08401-2 (PMC8900370; doi:10.1186/s12864-022-08401-2)

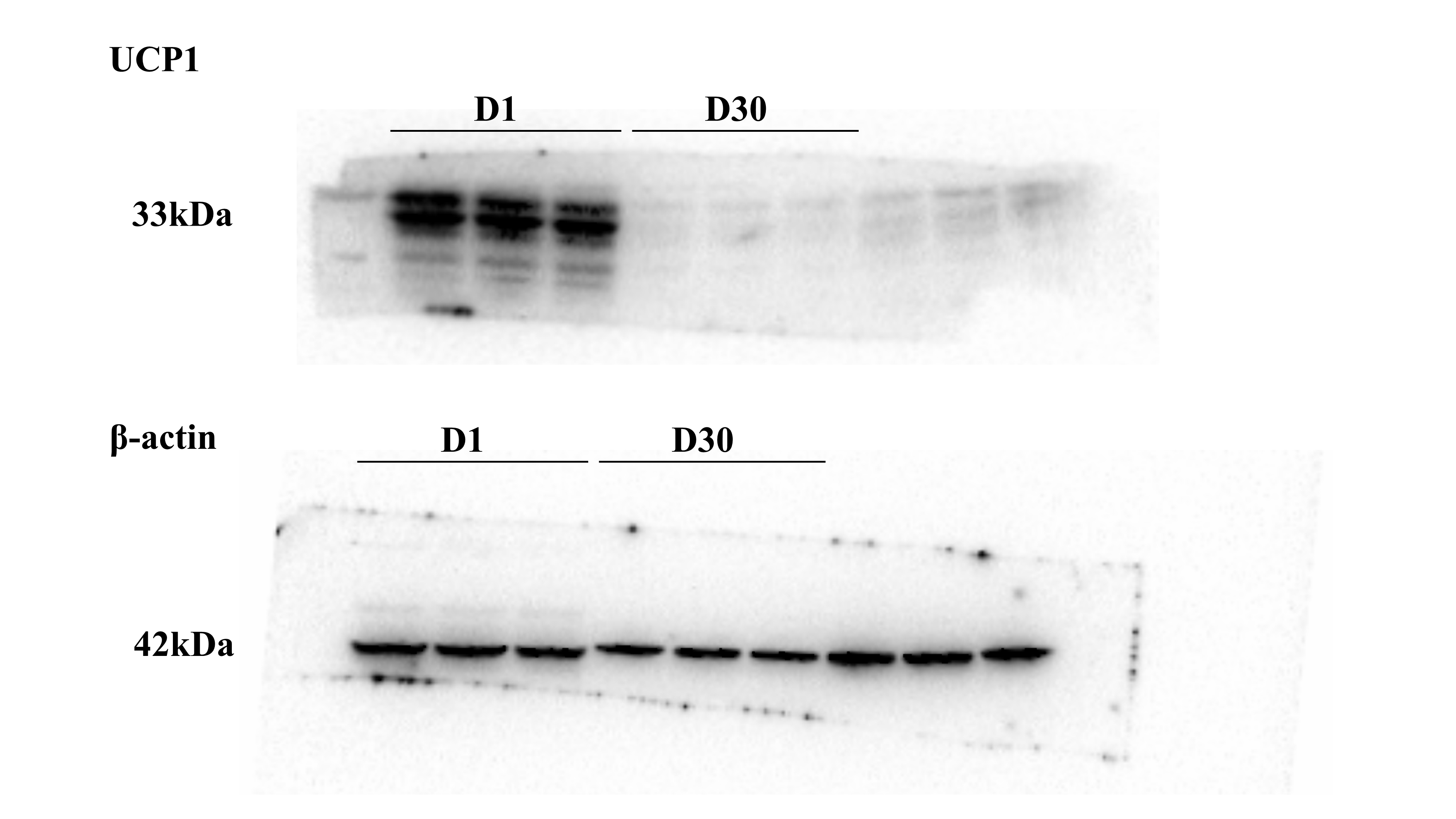

Supplement: Supplementary file 1 — Additional file 1: Fig. S1. Whole membrane images for Fig. 1C. [file 12864_2022_8401_MOESM1_ESM.tif]
