# Supplementary material for: RNA-Seq reveals miRNA role in thermogenic regulation in brown adipose tissues of goats
Source: BMC Genomics. 2022 Mar 7;23:186. doi: 10.1186/s12864-022-08401-2 (PMC8900370; doi:10.1186/s12864-022-08401-2)
Supplement: Supplementary file 2 — Additional file 2: Table S1. Sequencing data statistics. [file 12864_2022_8401_MOESM2_ESM.docx]

**Table S1. Sequencing data statistics**

| Sample | Raw reads | Low quality | Containing' N' reads | Length<18 | Length>30 | Clean reads | Q30 (%) |
| --- | --- | --- | --- | --- | --- | --- | --- |
| D1-1 | 26141402 | 0 | 203 | 5800001 | 1962753 | 18378445 | 98.85 |
| D1-2 | 18851943 | 0 | 140 | 4068866 | 1536118 | 13246819 | 99.06 |
| D1-3 | 23732695 | 0 | 158 | 3379436 | 1962186 | 18390915 | 99.15 |
| D30-1 | 19805978 | 0 | 139 | 1805466 | 507195 | 17493178 | 99.17 |
| D30-2 | 20558672 | 0 | 145 | 2764145 | 1175017 | 16619365 | 99.11 |
| D30-3 | 21020729 | 0 | 177 | 948562 | 543563 | 19528427 | 99.2 |
