# Supplementary material for: RNA-Seq reveals miRNA role in thermogenic regulation in brown adipose tissues of goats
Source: BMC Genomics. 2022 Mar 7;23:186. doi: 10.1186/s12864-022-08401-2 (PMC8900370; doi:10.1186/s12864-022-08401-2)
Supplement: Supplementary file 3 — Additional file 3: Table S2. Statistical table of sRNA classification annotation. [file 12864_2022_8401_MOESM3_ESM.docx]

**Table S2. Statistical table of sRNA classification annotation**

| Sample | rRNA | scRNA | snRNA | snoRNA | tRNA | Repbase | Unannotated |
| --- | --- | --- | --- | --- | --- | --- | --- |
| D1-1 | 2039741 | 0 | 12 | 49802 | 92997 | 433199 | 15762694 |
| D1-2 | 1050216 | 0 | 6 | 50764 | 70648 | 245172 | 11830013 |
| D1-3 | 1106672 | 0 | 11 | 53394 | 97455 | 230995 | 16902388 |
| D30-1 | 314237 | 0 | 8 | 26738 | 24114 | 54970 | 17073111 |
| D30-2 | 361996 | 0 | 8 | 21717 | 29403 | 63144 | 16143097 |
| D30-3 | 319925 | 0 | 18 | 41949 | 32457 | 52550 | 19081528 |
