# Supplementary material for: RNA-Seq reveals miRNA role in thermogenic regulation in brown adipose tissues of goats
Source: BMC Genomics. 2022 Mar 7;23:186. doi: 10.1186/s12864-022-08401-2 (PMC8900370; doi:10.1186/s12864-022-08401-2)
Supplement: Supplementary file 4 — Additional file 4: Table S3. Comparison information of reference genome. [file 12864_2022_8401_MOESM4_ESM.docx]

**Table S3. Comparison information of reference genome**

| Sample | Total Reads | Mapped Reads | Mapped reads(+) | Mapped reads(-) |
| --- | --- | --- | --- | --- |
| D1-1 | 15,762,694 | 9,801,225  （62.18%） | 5,452,210  （34.59%） | 4,349,015  （27.59%） |
| D1-2 | 11,830,013 | 7,403,297  （62.58%） | 4,230,431  （35.76%） | 3,172,866  （26.82%） |
| D1-3 | 16,902,388 | 10,833,790  （64.10%） | 5,836,696  （34.53%） | 4,997,094  （29.56%） |
| D30-1 | 17,073,111 | 11,102,376  （65.03%） | 5,434,220  （31.83%） | 5,668,156  （33.20%） |
| D30-2 | 16,143,097 | 10,944,061  （67.79%） | 5,662,315  （35.08%） | 5,281,746  （32.72%） |
| D30-3 | 19,081,528 | 11,754,534  （61.60%） | 5,662,238  （29.67%） | 6,092,296  （31.93%） |
