# Supplementary material for: RNA-Seq reveals miRNA role in thermogenic regulation in brown adipose tissues of goats
Source: BMC Genomics. 2022 Mar 7;23:186. doi: 10.1186/s12864-022-08401-2 (PMC8900370; doi:10.1186/s12864-022-08401-2)
Supplement: Supplementary file 10 — Additional file 10: Table S9. Sequences of primer used for RT-qPCR. [file 12864_2022_8401_MOESM10_ESM.docx]

**Table S9. Sequences of primer used for RT-qPCR**

| Gene | Forward primer | Reverse primer | Tm(°C) | Size(bp) |
| --- | --- | --- | --- | --- |
| UCP1 | TCCTGTCTTTGATCGCCTCT | GAACAGTCCATGTGCCAGTG | 61.3 | 135 |
| PGC1α | CCACAAATGATGACCCTC | GGTTTGGCTTGTAGATGTT | 60 | 103 |
| PPARγ | GTGTCACTCCTGAACGAAAT | GGAAATGCTGGAGAAGTCAA | 60 | 156 |
| FABP4 | ACTGGGATGGGAAATCAACC | CCTTGGCTTATGCTCTCTCG | 60 | 117 |
| FASN | CTGCTCAGTGGGCTCCTCA | TGGCGGTCAGTGGCTATGT | 60 | 187 |
| COX1 | GAGCCCCCGACATAGCATTT | GCTCCTGCATGGGCTAGATT | 56.9 | 160 |
| ATP6 | TTAGGCCTTCTACCCCACTCA | GGATTAGTGGTGTGGGCGTT | 53.7 | 163 |
| TBP | TCGCCAAGAATAGTGTGCTG | CCGTAAGGCATCATTGGACT | 61.3 | 202 |
| U6 | GGAACGATACAGAGAAGATTAGC | TGGAACACTTCTGGAATTTGCA | 60 | 68 |
